# Time to Move Beyond a “One-Size Fits All” Approach to Inspiratory Muscle Training

**DOI:** 10.3389/fphys.2021.766346

**Published:** 2022-01-10

**Authors:** Ren-Jay Shei, Hunter L. Paris, Abigail S. Sogard, Timothy D. Mickleborough

**Affiliations:** ^1^Global Medical Department, Mallinckrodt Pharmaceuticals Company, Hampton, NJ, United States; ^2^Department of Sports Medicine, Pepperdine University, Malibu, CA, United States; ^3^Department of Kinesiology, School of Public Health-Bloomington, Indiana University, Bloomington, IN, United States

**Keywords:** training prescription, performance, respiratory muscle strength, respiratory muscle endurance, pulmonary function

## Abstract

Inspiratory muscle training (IMT) has been studied as a rehabilitation tool and ergogenic aid in clinical, athletic, and healthy populations. This technique aims to improve respiratory muscle strength and endurance, which has been seen to enhance respiratory pressure generation, respiratory muscle weakness, exercise capacity, and quality of life. However, the effects of IMT have been discrepant between populations, with some studies showing improvements with IMT and others not. This may be due to the use of standardized IMT protocols which are uniformly applied to all study participants without considering individual characteristics and training needs. As such, we suggest that research on IMT veer away from a standardized, one-size-fits-all intervention, and instead utilize specific IMT training protocols. In particular, a more personalized approach to an individual’s training prescription based upon goals, needs, and desired outcomes of the patient or athlete. In order for the coach or practitioner to adjust and personalize a given IMT prescription for an individual, factors, such as frequency, duration, and modality will be influenced, thus inevitably affecting overall training load and adaptations for a projected outcome. Therefore, by integrating specific methods based on optimization, periodization, and personalization, further studies may overcome previous discrepancies within IMT research.

## Introduction

Inspiratory muscle training (IMT) has been thoroughly investigated over several decades as a rehabilitation tool (Gosselink et al., 2011; Smart et al., 2013; Cahalin and Arena, 2015; Menezes et al., 2016; Shei et al., 2016b; Charususin et al., 2018a; Shei and Mickleborough, 2019) and ergogenic aid (Sheel, 2002; Illi et al., 2012; HajGhanbari et al., 2013; Karsten et al., 2018; Shei, 2018) in healthy, clinical, and athletic populations, with generally positive findings. IMT is an intervention aimed to strengthen the inspiratory muscles, primarily the diaphragm and other inspiratory muscles such as the external intercostals, scalenes, and sternocleidomastoid (Celli, 1986; Sheel, 2002; Ratnovsky and Elad, 2005; Dominelli and Sheel, 2012; Illi et al., 2012; HajGhanbari et al., 2013; Shei et al., 2016b; Reid et al., 2018; Walterspacher et al., 2018; Ando et al., 2020; Derbakova et al., 2020). In clinical populations, IMT may aid in overcoming disease-associated pathologies related to the pulmonary system, such as respiratory muscle weakness, altered operating lung volumes, and expiratory flow limitation, thus improving clinical status, exercise capacity, and quality of life (Aaron et al., 1992a,b; Lisboa et al., 1994, 1997; Johnson et al., 1995; Kosmas et al., 2004; Calverley and Koulouris, 2005; Dhand, 2005; McConnell, 2005; Turner et al., 2012; Laviolette et al., 2014; Price et al., 2014; Weatherald et al., 2017; Moore et al., 2018; Chung et al., 2021). Conversely, in healthy and athletic populations, IMT can enhance respiratory muscle function, translating into a potential ergogenic benefit even in the absence of pulmonary system abnormalities. Several seminal studies have documented significant respiratory muscle fatigue during exercise (Johnson et al., 1992; Harms et al., 1997, 1998; St Croix et al., 2000) and observed a respiratory muscle metaboreflex (Harms et al., 1997, 1998; Witt et al., 2007). The respiratory muscle metaboreflex is a phenomenon where blood is shunted away from locomotor muscles and toward respiratory muscles in response to a large increase in the work of breathing (Dominelli et al., 2017; Sheel et al., 2018). More recently, the role of the respiratory muscles during exercise and the occurrence of respiratory muscle fatigue during and after exercise has been an area of focus (Dempsey et al., 2006, 2012; Aliverti, 2016; Oueslati et al., 2018). Thus, even in non-clinical populations, IMT may enhance respiratory muscle function.

**FIGURE 1:**
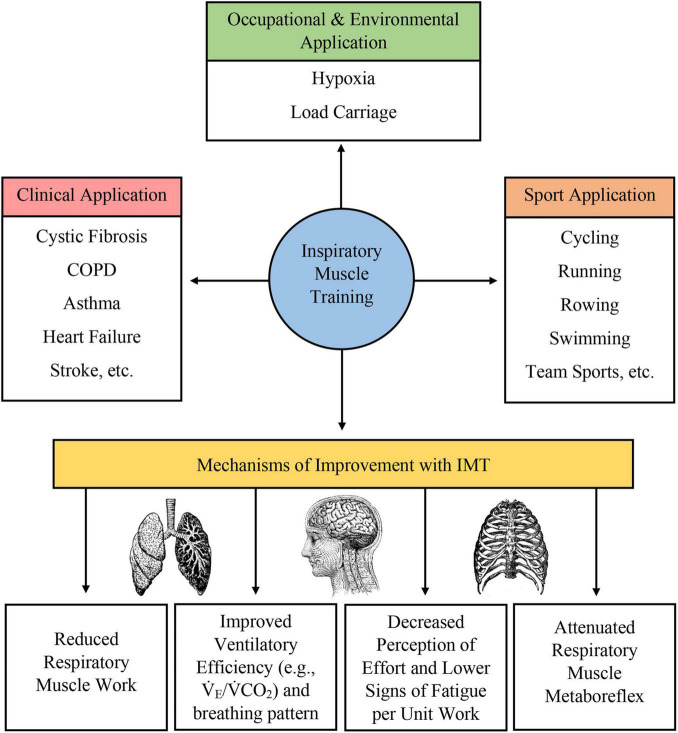
Summary of clinical, occupational, environmental, and sport application of IMT as well as a brief overview of physiological adaptations induced by IMT.

Many different forms of IMT have been developed, including pressure-based and volume-based loading protocols. Typically, these protocols require subjects to inspire against a resistance or maintain a prescribed level of minute ventilation to load the respiratory muscles and produce a training adaptation. IMT (1) promotes diaphragm hypertrophy (Enright et al., 2006; Downey et al., 2007; Shei, 2018; Shei et al., 2018); (2) increases the proportion of type I fibers and the size of type II fibers in the external intercostal muscles (Huang et al., 2003); (3) attenuates the respiratory muscle metaboreflex (Sheel, 2002; Gething et al., 2004; Enright et al., 2006; Witt et al., 2007; McConnell, 2009; Turner et al., 2012, 2016; Raux et al., 2013, 2016; Price et al., 2014; Lomax et al., 2017); (4) decreases inspiratory muscle motor drive with preserved pressure generation (Huang et al., 2003; Price et al., 2014; Ramsook et al., 2017); (5) improves respiratory muscle economy (Enright et al., 2006; Turner et al., 2012; Held and Pendergast, 2014; Shei et al., 2016a,b; Shei, 2018); 6) decreases the rating of perceived breathlessness or rating of perceived exertion (Sheel, 2002; Gething et al., 2004; Downey et al., 2007; McConnell, 2009; Price et al., 2014; Lomax et al., 2017; Ramsook et al., 2017); (7) reduces the work of breathing (Gething et al., 2004; McConnell, 2009; Turner et al., 2012; Price et al., 2014; Shei et al., 2016a,b); (8) improves respiratory muscle endurance (Gething et al., 2004; Enright et al., 2006; McConnell, 2009; Price et al., 2014; Sales et al., 2016; Shei et al., 2016a,b); (9) improves ventilatory efficiency (Sheel, 2002; Gething et al., 2004; Enright et al., 2006; Turner et al., 2012; Bernardi et al., 2014; Price et al., 2014; Lomax et al., 2017; Salazar-Martínez et al., 2017); (10) reorganizes respiratory muscle recruitment pattern (Enright et al., 2006; Walterspacher et al., 2018); (11), improves breathing pattern during exercise hyperpnea (Charususin et al., 2016); and (12) reduces cytokine release (Mills et al., 2013, 2014). While some of these adaptations are well-characterized, others are postulated to occur with published studies showing conflicting results. For example, while Ray et al. (2010) observed a reduction in work of breathing following IMT, Langer et al. (2018) did not. Putatively, not all these proposed adaptations may be observed in all populations who undertake IMT, and population-specific and individual-specific variations could reasonably be expected. Collectively, these adaptations may underpin exercise enhancement or functional improvement in athletic and clinical populations. More recently, IMT has been studied in occupational settings, such as military and emergency services and recreational settings, which require personnel and participants to exercise while carrying a load on the thoracic cavity (e.g., protective equipment, backpacks to transport gear and provisions, etc.) (Sperlich et al., 2009; Faghy et al., 2016; Shei et al., 2017, 2018; Shei, 2018; Hinde et al., 2020). In this application, IMT appears to be effective in improving work and exercise capacity, and is likely due, in part, to the higher ventilatory demand and workload due to load carriage. Thus, enhancements in respiratory muscle function here again optimize performance. A summary of physiological adaptation to IMT and applications of IMT is given in Figure 1.

## Inconsistencies in Inspiratory Muscle Training

In spite of these findings, translation of physiological adaptations into clinically or competitively meaningful improvements for clinical and athletic populations has not been uniformly observed (McConnell, 2012; Patel et al., 2012). Contributing factors to these heterogeneous findings likely include variations in the study population, study sample size, training protocol (intensity, duration, frequency, rest intervals, etc.), whether training was completed at rest or during concurrent exercise, training type (pressure-threshold, flow-resistive, normocapneic hyperpnea, etc.), among other factors (Illi et al., 2012; Patel et al., 2012; HajGhanbari et al., 2013; Formiga et al., 2018; Shei, 2018; Larribaut et al., 2020). A separate consideration is the ability to reliably evaluate respiratory muscle functional outcomes, such as strength, endurance, activation pattern, etc., to appreciate the potential benefits associated with respiratory muscle training. Most commonly, maximal inspiratory and expiratory mouth pressures (*P*I_max_, *P*E_max_, respectively) are used to assess respiratory muscle strength. Other measures, including but not limited to transdiaphragmatic pressure, sustained maximal inspiratory pressure (SMIP), respiratory muscle power output, fatigue index, inspiratory duty cycle, minute ventilation, breathing frequency, and tidal volume, have also been used to assess respiratory muscle function. Presently however, there is no consensus as to which measures are most appropriate in the context of evaluating responses to IMT. While it is reasonable to tailor outcome measures to the stated goals of each study, developing a core set of common measures would be a prudent step forward in enhancing the rigor of future IMT studies.

Several recent reviews concluded that matching the IMT training prescription to the ventilatory demands of the exercise task likely optimize the ergogenic effect of IMT (HajGhanbari et al., 2013; Shei, 2018). This recommendation for IMT training specificity highlights the need to critically examine the training protocols which have been tested and to evaluate whether the training load imposed by these protocols sufficiently overloads the respiratory muscle to induce meaningful training adaptations (Formiga et al., 2018; Larribaut et al., 2020). Indeed, the concept of training specificity, in which the training stimulus is matched as closely as possible to the criterion task, has long been understood and adopted in the field of sport science (Hawley, 2008). Yet, the majority of IMT research has used fixed protocols, some of which were developed for clinical populations (Johnson et al., 1998; Villafranca et al., 1998; Weiner et al., 1999, 2000) but broadly applied to healthy and athletic populations. One of the most common pressure-threshold IMT protocols involves completing 30 breaths, twice daily at 50% of a subject’s *P*I_max_, five times a week for 6 weeks (Romer et al., 2002a,b; Kilding et al., 2010; Turner et al., 2012). While many variations exist and prescribe varying numbers of repetitions, frequencies, and durations, IMT training “intensities” are still largely based on a percentage of *P*I_max_ or SMIP. Acknowledging that for research purposes, standardizing intervention protocols is necessary for experimental control and uniformity between subjects, from a practitioner’s standpoint, this approach may not be optimal for producing *training* adaptations in real-world settings. As such, we propose that further consideration be given to the *training* aspect of IMT.

## Training Prescription Considerations

Coaches and practitioners routinely tailor training programs to suit the goals and needs of individual athletes and constantly make adjustments based on numerous factors, including the athlete’s response to training, injuries, illnesses, and environmental factors, to name a few. The training prescription is seldom uniform and typically contains varied workouts, commonly organized into micro-, meso-, and mega-cycles in a periodized fashion (Issurin, 2010; Kiely, 2018; Bompa and Buzzichelli, 2019). In this light, perhaps it is time to investigate IMT as a training tool, rather than a research intervention, and consider periodization of IMT and varying individual training sessions to achieve a specific training goal (aerobic, threshold, neuromuscular, anaerobic, etc.) much like sports training varies workout prescription to achieve training adaptations. In order to achieve this, particular consideration will need to be given to the context or application of IMT, i.e., rehabilitation vs. ergogenic aid for sport performance. Within these contexts, factors, such as frequency, duration, and modality (which affect overall training load) will influence how the coach or practitioner adjusts the given IMT prescription.

Training load is perhaps the most important factor in optimizing IMT prescription. While historic protocols are somewhat individualized in that the “intensity” prescribed is based on an individual’s *P*I_max_ or SMIP, the percentage of *P*I_max_ or SMIP is often arbitrarily selected and then fixed at that level. Careful consideration of the training goals, such as improving respiratory muscle strength vs. endurance, might lead one to prescribe a higher “intensity” for a strength-oriented training session or a lower “intensity” for endurance-oriented sessions, to allow a subject to complete more repetitions or a longer training session. Within training load, the number of repetitions could also be adjusted according to the goals of an individual training session. Special consideration should be given for training toward a pre-defined “task failure,” as some studies have shown that in limb locomotor resistance training, a larger training effect is produced when training is conducted to task failure (Burd et al., 2010; Schoenfeld et al., 2015, 2016). As previously suggested, in the context of IMT, task failure may be defined by using a pre-specified threshold of inspiratory pressure, volume, or minute ventilation, or failure to achieve a prescribed breathing frequency, tidal volume, or both (Shei, 2020). Using a pre-defined task failure threshold could help ensure that each training session provides a sufficient training stimulus to induce adaptations which in turn may enhance the efficacy of IMT. This is particularly important for populations requiring a higher training load to produce training adaptations in the respiratory muscles, such as swimmers and other aquatic-based athletes, including SCUBA divers (Lindholm et al., 2007; Mickleborough et al., 2008; Shei et al., 2016a,b; Vašíčková et al., 2017; Lomax et al., 2019; Yañez-Sepulveda et al., 2021). Moreover, this approach could help match the IMT training prescription to the ventilatory demands of the athlete or patient’s goals, whether that is sustained hyperpnea during prolonged exercise or improving the ability to complete activities of daily living.

Aside from the broader considerations of training frequency, intensity, and periodization, critically evaluating the specific training mode will also be crucial to optimizing future IMT prescription and research. Several recent areas of investigation have garnered growing interest, which merits further discussion. First, there is increasing interest in concurrent exercise and IMT, i.e., rather than completing IMT at rest, the athlete or patient uses the IMT device while simultaneously completing another exercise, such as running or cycling (Hellyer et al., 2015; Granados et al., 2016; McEntire et al., 2016; Porcari et al., 2016; Shei, 2018; Shei et al., 2018). To date, only a small number of studies have investigated concurrent IMT and exercise. However, early findings suggest that IMT performed during concurrent cycling exercise results in greater diaphragm activation, as demonstrated by electromyography (EMG), and that concurrent training improves both ventilatory threshold and respiratory compensation threshold, and power output at these thresholds (Hellyer et al., 2015; Porcari et al., 2016). Using an IMT device during exercise may cause mild hypoxemia, possibly due to inadequate hyperventilation (Granados et al., 2016). Factors, such as the breathing pattern that can be sustained against the external resistance may influence whether exercise hyperpnea with concurrent IMT may be adequate. It is also possible that a relative hypoventilation relative to work rate could lead to a decrease in work rate intensity to preserve arterial blood gases, rather than causing hypoxemia. Relative hypoventilation in this context may also induce hypercapnia, which could limit performance but also represent an additional training stimulus. Considering these factors, it is certainly possible that concurrent IMT and exercise may compromise endurance exercise performance and workload during a given training session. Thus, any compromise in the ability to sustain a given workload resulting from simultaneously using an IMT device should be weighed against the potential benefits of loading the respiratory muscles with the IMT device. As previously discussed, even in this context, consideration should be given to the selected load and duration of training for concurrent IMT and exercise. A recent study employing an inspiratory load of 15% of *P*I_max_ concurrently during exercise training found no difference between concurrent IMT plus exercise training and exercise training alone after 3 weeks of training. After 6 weeks of training, however, the concurrent training produced an ∼8% improvement 5-mile cycling time trial performance (McEntire et al., 2016). Therefore, even given a relatively low resistive load, concurrent IMT and exercise training over a longer period may still induce appreciable respiratory muscle adaptations and subsequent performance benefits.

Next, the lung volume(s) at which IMT is completed are also important to consider, as highlighted by a recent publication by Van Hollebeke et al. (2020). In this study, 48 healthy volunteers were randomly assigned to perform either pressure-threshold IMT initiated from residual volume (RV) or functional residual capacity (FRC), or tapered flow resistive loading initiated from RV. The authors found that only training initiated from FRC resulted in consistent improvements in respiratory muscle function at higher lung volumes, whereas improvements after the standard protocol initiated from RV were restricted to PI_max_ gains at lower lung volumes. Thus, considering the operating lung volumes of the athlete or patient’s activities may be an important factor when deciding the lung volumes at which IMT should be initiated.

Finally, work led by Dominelli et al. (2015a,b), Molgat-Seon et al. (2018a,b), Welch et al. (2018a,b), Geary et al. (2019), and Archiza et al. (2021) has shown apparent sex differences in respiratory muscle fatigability and workload, which is important to consider in the context of IMT. Due in part to anatomical differences, such as smaller airway diameter and smaller thoracic volume compared to males, females generally have a higher work of breathing when minute ventilation, operating lung volume, breathing frequency, and tidal volume are matched (Dominelli et al., 2019). More recent evidence suggests that females are more resistant to respiratory muscle fatigue compared with males (Welch et al., 2018a; Geary et al., 2019), although this difference in respiratory muscle fatigability does not appear to influence exercise performance (Welch et al., 2018b). Despite this, because of the comparatively higher respiratory muscle fatigue resistance in females, it may be that females require a higher prescribed IMT training load compared with males in order to induce training adaptations. Interestingly, however, a recent investigation of respiratory muscle endurance training (RMET) in healthy active men and women found a greater ergogenic effect of RMET on cycling time trial performance in women compared to men, an effect which was even more pronounced in hypoxia (Chambault et al., 2021). It is possible then, that females may not in fact require a higher prescribed training workload, and that there may in fact be a sex-specific differential response to RMET. Regardless, further investigation on sex differences in both IMT prescription and response is warranted.

Most respiratory muscle training paradigms have focused on IMT in patients with pulmonary disease [e.g., asthma, chronic obstructive pulmonary disease (COPD)] or respiratory muscle weakness (e.g., multiple sclerosis, Parkinson’s disease) with expectations to improve ventilatory capacity. While expiration during resting is passively mediated by the recoil of the lung and thorax, forced expiration and expiration during exercise requires expiratory muscle activation, which requires muscles of the abdominal wall, in particular the transverse abdominis and the internal and external oblique muscles, as well as internal intercostals. Expiratory muscles, especially the upper airway musculature, play an essential role during phonation, airway clearance and expectoration. Therefore, interest in expiratory muscle strength training (EMT) has developed, particularly for improving non-ventilatory functions, such as coughing, speaking, and swallowing. A number of studies have shown that EMT is effective in increasing the strength of the expiratory muscles resulting in augmenting the expiratory driving pressure used for cough, speech, or swallow (Kim and Sapienza, 2005). EMT elicits similar responses to IMT in the expiratory muscle system, and similar to IMT, improvement of the maximal expiratory pressure is the hallmark parameter of effective EMT. Interestingly, EMT leads to improved maximal inspiratory pressure, indicating involvement of the inspiratory muscles in the process of expiration, whereas IMT does not improve maximal expiratory pressure (McConnell, 2013).

While the ergogenic benefits of either IMT or EMT alone have clearly been established, combined IMT/EMT have not been widely reported. However, a number of studies have highlighted the possibly overlooked ergogenic potential of combined IMT/EMT in patients with Duchenne muscular dystrophy or spinal cord muscular atrophy (Gozal and Thiriet, 1999), multiple sclerosis (Ray et al., 2013) and COPD (Weiner et al., 2003). These, and other studies, indicate that combined IMT/EMT may at least be equally effective to either method alone, and might be the preferred method of RMT in respiratory muscle disorders in which training of both muscle groups is of greater importance, such as in COPD and neuromuscular disorders.

A few studies have investigated the effect of combined IMT/EMT on respiratory muscle function and exercise performance in healthy individuals. Griffiths and McConnell (2007) showed that 4 weeks of IMT and EMT increased inspiratory and expiratory mouth pressures, respectively. However, only IMT improved rowing performance, while EMT and combined IMT/EMT did not improve rowing performance (Griffiths and McConnell, 2007). Amonette and Dupler (2002) using combined IMT/EMT for 4 weeks showed that this type of training, although increasing strength of the respiratory muscles as seen by increases in expiratory mouth pressures, produced no changes in pulmonary function or VO_2max_ (Amonette and Dupler, 2002). However, this study was limited by a low sample size (eight subjects in the treatments group and four subjects in the control group).

## Future Directions and Challenges

**FIGURE 2:**
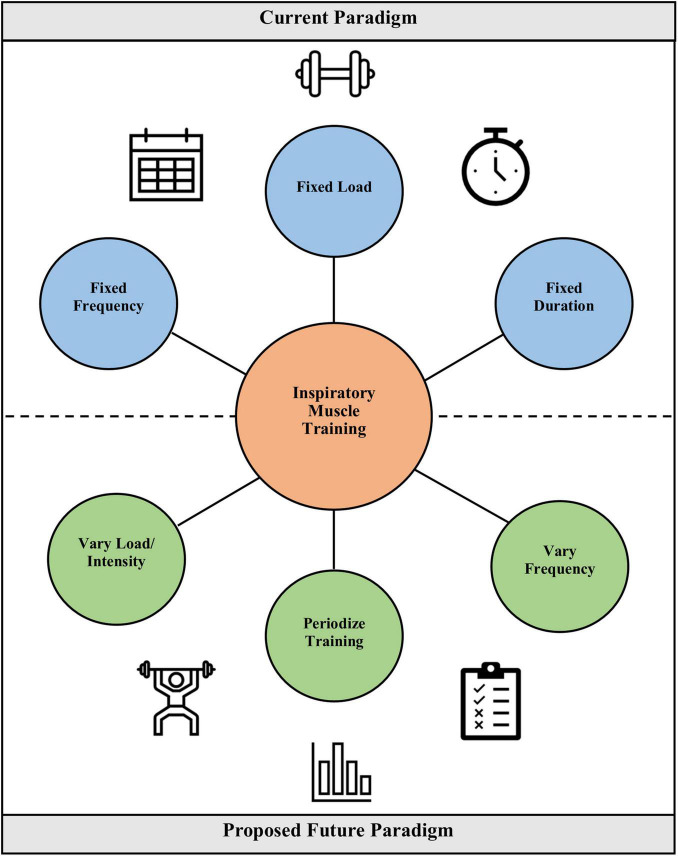
Comparison of the current paradigm for IMT prescription vs. a proposed future paradigm in which load and intensity are varied, training is periodized, and frequency is varied. The latter constitutes a more individualized training prescription akin to how sport coaches prescribe training to their athletes.

Future studies in IMT should aim to address the considerations discussed here, and in particular, consider a more personalized or “precision” approach to tailoring training prescription to the individual needs and goals of the patient or athlete (Figure 2). Inherent in specifying treatment objectives is distinguishing between athletes and clinical populations. Whereas, athletes may seek to improve ventilatory efficiency or lessen diaphragmatic fatigue, the aim for COPD patients may be to lessen dyspnea or improve strength of the inspiratory muscles. While it will certainly be challenging to achieve an individualized approach while retaining adequate experimental control, developing and validating innovative methodologies better suited to individualized training will aid in determining whether such an approach is feasible and effective. Such approaches might use targeted training intensities for a fixed or variable period of time, and the timing of progression to different intensities could be dictated by a pre-defined, but uniformly applied protocol. These “checkpoints” for progression could be based on changes in *P*I_max_, the ability to complete a given set of training breaths, or the ability to complete a progressive test, such as the test of incremental respiratory endurance (TIRE) regimen. The TIRE regimen is a common protocol in flow-resistive IMT, such as with the RT2 and PrO_2_ devices (Mickleborough et al., 2008; Shei et al., 2016a,^2018^; Hursh et al., 2019). Similarly, training groups could be enrolled at similar points in their training cycles to adapt a periodized approach to IMT into research studies and real-world application. These novel approaches will require testing and validation, but should they bear fruit, these innovations can help usher in a new era of IMT research that addresses fundamental questions regarding how to optimize training prescription and the consequent adaptations and benefits.

Aside from adjusting training protocols, future studies also need to consider what endpoints are most relevant, how to adequately power and control studies (for example, achieving a proper placebo control and double-blinding studies), how to balance baseline participant characteristics, what sample size is needed, and how to design and execute multicenter studies. As highlighted by Patel and colleagues (Patel et al., 2012), a large, randomized, placebo-controlled, double-blinded, multicenter, akin to what a Phase 3 pivotal trial would be for pharmacological interventions, would be a significant step forward in elucidating and validating any potential efficacy resulting from IMT. Recognizing that even within homogenous groups responders and non-responders appear, large-scale projects should also be paired with methodological designs equipped to analyze individual responses—which assumes individual variability will be both present and relevant—as has been demonstrated recently (Hecksteden et al., 2018). While such studies are costly, complex, and require a robust network of qualified investigators, the data from such trials have become the standard for determining the safety and efficacy of medical interventions.

To the best of the authors’ knowledge, only three large randomized controlled trials studying IMT have been completed, all in the COPD patient population (Beaumont et al., 2018; Charususin et al., 2018b; Schultz et al., 2018). However, while these studies enrolled 611 (Schultz et al., 2018), 219 (Charususin et al., 2018b), and 150 patients (Beaumont et al., 2018) patients respectively, they were all single-center studies. All three studies found that IMT enhanced respiratory muscle function, and while one study found reductions in dyspnea symptom scores during endurance cycling (Charususin et al., 2018b), the other two found no benefit of IMT on quality of life or dyspnea (Beaumont et al., 2018; Schultz et al., 2018), and none of the studies observed improvements in 6-min walk distance. These studies suggest that there may not be a positive benefit from IMT. In contrast, however, a smaller study by some of the same authors of the large, multicenter randomized controlled trials found that IMT in COPD patients with low maximal inspiratory pressures did improve dyspnea and exercise endurance, which was associated with a reduced diaphragm activation relative to maximum (Langer et al., 2018). Discrepant findings between these three studies may be due to a number of factors including the baseline characteristics of the patients, whether physiological and perceptual improvements (respiratory muscle function, dyspnea, etc.) translate into functional improvements, whether the training protocol provided an adequate training stimulus, and whether training was sustained for a sufficient time. A key difference between the three large studies (Beaumont et al., 2018; Charususin et al., 2018b; Schultz et al., 2018) and the smaller study (Langer et al., 2018) is how the intervention and control interventions were provided. In the larger studies the intervention was general exercise training plus IMT, and control was general exercise training plus sham IMT, whereas the smaller study investigated the effect of IMT as a standalone intervention vs. sham IMT. The second difference is that the constant work rate endurance test was used as an outcome in smaller study. Another factor that is frequently overlooked is that “sham” IMT against relatively low resistances could have effects on respiratory muscle function especially in frail populations, such as many older patients with chronic diseases or patients admitted to the ICU. Thus, questions remain regarding whether more nuanced and progressive individualized training prescriptions may produce different outcomes given the physiological plausibility behind putative IMT benefits. Perhaps then, it is time to consider adopting new approaches to IMT research to tailor the right intervention to the right population and optimize treatment/training effects, and determine whether there is, or is not, a true benefit of IMT.

## Conclusion

In summary, despite decades of research on IMT, with some studies showing clear benefit and others showing no benefit, it is uncertain why some populations respond to IMT, and some do not. While there are certainly inherent differences in study populations and how IMT is being applied (i.e., as a rehabilitative tool, or for endurance exercise, or team sport exercise), questions regarding how training prescription has historically been done and whether that approach truly optimizes the response to IMT remain. It is time to consider new approaches to IMT that better match how practitioners in sport and exercise training design and apply training plans for athletes. By integrating these methods, such as periodization, better optimization of training load, and considering other factors, such as concurrent IMT and exercise training or the lung volumes at which IMT is completed, future studies may overcome previous shortcomings by providing a tailored, personalized approach that addresses the needs of the individual athlete or patient.

## Data Availability Statement

The original contributions presented in the study are included in the article/supplementary material, further inquiries can be directed to the corresponding author.

## Author Contributions

R-JS conceived of the idea. R-JS, HP, AS, and TM drafted and critically revised the manuscript. All authors reviewed and approved the final version of the manuscript prior to submission.

## Conflict of Interest

R-JS is an employee of Mallinckrodt Pharmaceuticals. The work described herein is solely reflective of the author’s personal views and is unrelated to his job duties with Mallinckrodt Pharmaceuticals. These views do not constitute an endorsement by Mallinckrodt Pharmaceuticals, do not represent the views of Mallinckrodt Pharmaceuticals, and Mallinckrodt Pharmaceuticals had no role in the conception, writing, revision, or final approval of the manuscript. The author may or may not hold stock in Mallinckrodt Pharmaceuticals, and does not have any other financial relationships or conflicts to disclose. The remaining authors declare that the research was conducted in the absence of any commercial or financial relationships that could be construed as a potential conflict of interest.

## Publisher’s Note

All claims expressed in this article are solely those of the authors and do not necessarily represent those of their affiliated organizations, or those of the publisher, the editors and the reviewers. Any product that may be evaluated in this article, or claim that may be made by its manufacturer, is not guaranteed or endorsed by the publisher.
